# Neuronal activity mediated regulation of glutamate transporter GLT‐1 surface diffusion in rat astrocytes in dissociated and slice cultures

**DOI:** 10.1002/glia.22997

**Published:** 2016-05-17

**Authors:** Sana Al Awabdh, Swati Gupta‐Agarwal, David F. Sheehan, James Muir, Rosalind Norkett, Alison E. Twelvetrees, Lewis D. Griffin, Josef T. Kittler

**Affiliations:** ^1^ Department of Neuroscience, Physiology and PharmacologyUniversity College LondonUnited Kingdom; ^2^ Department of Computer ScienceUniversity College LondonUnited Kingdom

**Keywords:** synapse, neuron‐astrocyte interaction, single particle tracking, organotypic slices

## Abstract

The astrocytic GLT‐1 (or EAAT2) is the major glutamate transporter for clearing synaptic glutamate. While the diffusion dynamics of neurotransmitter receptors at the neuronal surface are well understood, far less is known regarding the surface trafficking of transporters in subcellular domains of the astrocyte membrane. Here, we have used live‐cell imaging to study the mechanisms regulating GLT‐1 surface diffusion in astrocytes in dissociated and brain slice cultures. Using GFP‐time lapse imaging, we show that GLT‐1 forms stable clusters that are dispersed rapidly and reversibly upon glutamate treatment in a transporter activity‐dependent manner. Fluorescence recovery after photobleaching and single particle tracking using quantum dots revealed that clustered GLT‐1 is more stable than diffuse GLT‐1 and that glutamate increases GLT‐1 surface diffusion in the astrocyte membrane. Interestingly, the two main GLT‐1 isoforms expressed in the brain, GLT‐1a and GLT‐1b, are both found to be stabilized opposed to synapses under basal conditions, with GLT‐1b more so. GLT‐1 surface mobility is increased in proximity to activated synapses and alterations of neuronal activity can bidirectionally modulate the dynamics of both GLT‐1 isoforms. Altogether, these data reveal that astrocytic GLT‐1 surface mobility, via its transport activity, is modulated during neuronal firing, which may be a key process for shaping glutamate clearance and glutamatergic synaptic transmission. GLIA 2016;64:1252–1264

## Introduction

Glutamate is the major excitatory neurotransmitter in the mammalian central nervous system affecting neuronal and glial function by acting on glutamate receptors. Maintaining the correct level of extracellular glutamate is crucial for neuronal transmission and network activity. Glutamate clearance is achieved by diffusion away from the synaptic cleft in conjunction with glutamate uptake by excitatory amino acid transporters (EAATs). These plasma membrane transporters use energy from the transmembrane ionic gradients to remove glutamate from the extracellular space (Attwell et al., 1993; Danbolt, 2001; Kanner and Schuldiner, 1987; Tzingounis and Wadiche, 2007). This glutamate uptake function allows EAATs to terminate and shape excitatory synaptic transmission, and prevent neuronal excitotoxicity (Rosenberg et al., 1992; Tzingounis and Wadiche, 2007).

EAAT2/GLT‐1 is highly expressed in the hippocampus and cortex and is the main astrocytic transporter involved in extracellular glutamate clearance in the adult forebrain (Chaudhry et al., 1995; Holmseth et al., 2012; Kojima et al., 1999; Ullensvang et al., 1997). Global knockout of GLT‐1 results in toxic increases in extracellular glutamate concentrations within the CNS, inducing lethal spontaneous seizures (Rothstein et al., 1996; Tanaka et al., 1997). Regulation of GLT‐1 expression, activity and trafficking modulates glutamate uptake and is implicated in plasticity (Omrani et al., 2009) and pathology (Danbolt, 2001; Petr et al., 2015; Tzingounis and Wadiche, 2007) making it a key target for new therapeutics (Soni et al., 2014). Two main isoforms of GLT‐1 are expressed in astrocytes in the adult brain, GLT‐1a and GLT‐1b (Chen et al., 2002, 2004; Holmseth et al., 2009; Sullivan et al., 2004), which differ only in their C‐terminal tails. Unlike GLT‐1a, the GLT‐1b isoform contains a PDZ binding domain that plays a role in its trafficking via interaction with PDZ domain containing scaffold proteins such as DLG1, PSD95, and PICK1 (Bassan et al., 2008; Gonzalez‐Gonzalez et al., 2008a; Underhill et al., 2015).

The molecular mechanisms that regulate GLT‐1 expression, trafficking, exocytosis and endocytosis are increasingly well understood (Gonzalez‐Gonzalez et al., 2008b; Kalandadze et al., 2002; Peacey et al., 2009; Rothstein et al., 2005; Shih et al., 2014; Susarla and Robinson, 2008; Underhill et al., 2015). In contrast, while it is now well established that the activity‐dependent regulation of neurotransmitter receptor surface diffusion dynamics in the neuronal membrane is a key mechanism for regulating synaptic signalling (Choquet and Triller, 2013; Groc et al., 2004; Levi et al., 2008; Muir et al., 2010), far less is known regarding the regulatory mechanisms of transporter surface diffusion in subdomains of the astrocyte membrane. Recently, it was shown that GLT‐1 surface diffusion can be increased by glutamate and that impairing GLT‐1 surface mobility impacted the kinetics of neuronal excitatory postsynaptic currents (Murphy‐Royal et al., 2015). However, whether GLT‐1 isoforms exhibit differential surface diffusion on the astrocyte membrane, and the consequences of neuronal activity alterations and neuronal firing on GLT‐1 surface clustering and mobility at individual activated synapses remain far less clear. Moreover, whether differences exist in the activity‐dependent regulation of GLT‐1 surface trafficking in astrocytes in dissociated culture compared to astrocytes *in situ* has also not been fully explored.

Here, using live cell imaging and fluorescence recovery after photobleaching (FRAP), we show that GLT‐1 can be found as clusters on the astrocyte surface that are rapidly dispersed upon glutamate treatment, dependent on GLT‐1 transporter activity. Interestingly, using single particle tracking, we find that both GLT‐1a and GLT‐1b, exhibit a more confined surface diffusion in regions of astrocyte processes proximal to synaptic sites, with the GLT‐1b isoform more stable in these regions than GLT‐1a. Additionally, we demonstrate that neuronal activity bidirectionally regulates the surface diffusion of both GLT‐1 isoforms providing a mechanism to increase GLT‐1 mobility at individual activated synapses. Finally, by imaging organotypic brain slices, we also show that GLT‐1 undergoes similar activity‐dependent regulation in astrocyte processes *in situ* in intact tissue. The activity‐dependent regulation of GLT‐1 surface diffusion may play a key role in locally regulating glutamate uptake and limiting glutamate spill over.

## Materials and Methods

### Plasmid Constructs

Rat GLT‐1a cDNA was N‐terminally tagged with EGFP by PCR cloning in frame into pEGFP‐C1 (Clontech) using primers (written 5′–3′) with the following N and C‐terminal GLT‐1a sequences, respectively: ATGGCATCAACCGAGGGTGC and TTATTTTTCACGTTTCCAAGGTTCTTCCTC. GLT‐1a and GLT‐1b are identical over the first 551 residues, differing only in the C‐termini. Thus, GLT‐1b was derived from the GLT‐1a cDNA using the same N‐terminal primer, but replacing the C‐terminal primer with one containing the GLT‐1b specific sequence (lowercase) as follows: tcatatgcaggtctcgatatccaggaatgggaaagg   TACCTTGCACTCATCTATTAC GACAGAG. V5 tags were introduced by PCR into GLT‐1a and 1b between two proline residues (P199 and P200) in the extracellular loop using the following primers: *cctgctgggcctggacagcacc*CCATCCGA GGAGGCCAATAC; *gggttggggatgggcttgcc*AGGTGCCACCAGAACT TTCT where lowercase text corresponds to the sequence of the V5 tag. GLT‐1a‐V5 plasmid was a kind gift from Rattray Lab (Peacey et al., 2009). Presynaptically targeted GCaMP5 (SyGCaMP5) was cloned using SyGCaMP2 (#26124) (Dreosti et al., 2009) from Addgene as a target vector and inserting GCaMP5G from Addgene (#31788) (Akerboom et al., 2012) via the restriction sites SalI and NotI.

### Preparation and Transfection of Astrocyte Cultures

Primary cultures of cortical astrocytes were prepared from E18 or P0 Sprague‐Dawley rats as previously described (Banker, 1998). Cells were maintained in Dulbecco's modified Eagle's medium DMEM GlutaMAX (Invitrogen) supplemented with 4.5 g/L glucose, 20% fetal bovine serum, 10 u/mL penicillinG, and 100 µg/mL streptomycin at 37°C with 5% CO_2_ in a humidified incubator. Media was exchanged the day after plating. Astrocytes were passaged when confluency was reached (10 days after plating). Astrocytes were transfected with Amaxa Nucleofector^®^ technology following the manufacturer's protocol.

### Preparation and Transfection of Mixed Culture and the Neuron‐Astrocyte Cocultures

Hippocampal cultures were obtained from E18 rat embryos as described previously with some modifications (Arancibia‐Carcamo et al., 2009). To enrich the culture with astrocytes, the neurons were kept 24 h after plating in attachment medium (Minimal Essential Medium, 10% Horse Serum, 1 mM Sodium Pyruvate and 0.6% Glucose) before replacing with maintenance medium (Neurobasal Medium, B27 supplement, Glutamax, 0.6% Glucose, Penstrep). Cells were transfected at 10 days *in vitro* (DIV10) by lipofectamine as previously described (Al Awabdh et al., 2012; Smith et al., 2014) and imaged at DIV13. After transfecting DIV7 hippocampal neurons with SyGCaMP5 (lipofectamine 2000), astrocytes were transfected by nucleofection with 4 µg of GLT‐1a‐V5 (Amaxa Nucleofector) and plated on top of the SyGCaMP5 transfected neurons (Stephen et al., 2015). Transfected astrocytes were maintained with neurons for 3 to 4 days before multi‐wavelength live‐imaging.

### Organotypic Hippocampal Slice Preparation and Transfection

Organotypic hippocampal brain slices were prepared following the Stopini interface method (Stoppini et al., 1991). Transverse brain slices (300 μm) were obtained from postnatal day 7 (P7) Sprague‐Dawley rats, using a vibratome (Leica VT1200 S) and put in ice‐cold dissection medium [HEPES buffered EBSS (Earle's Balanced Salt Solution)]. Slices were cultured on sterile 0.45 μm Omnipore membrane filters (Millipore) in a humidified incubator at 37°C with 5% CO2. Slices were maintained for at least 7 days in culture medium [72% MEM + glutamax (GIBCO), 25% HRS, supplemented with 20 mM HEPES, 36 mM glucose, and 1.06% Pen‐Strep (10 U mL^−1^, 100 µg ml^−1^) with 16% Nystatin (10,000 U ml^−1^) prior to transfection and imaged 3–6 days later. Media was changed the day after slicing and every three days after that. Organotypic slice cultures were biolistically transfected at 7 DIV using a Helios gene gun (BioRad; Stephen et al., 2015; Woods and Zito, 2008]. This involved coating small (0.6 µm) gold particles with up to 25 µg of GFP‐GLT‐1b‐V5. This allowed sparse transfection of astrocytes in organotypic slices (Benediktsson et al., 2012).

### FRAP Imaging and Analysis

Transfected astrocytes were perfused with imaging medium (10 mM HEPES pH = 7.4, 125 mM NaCl, 10 mM d‐Glucose, 5 mM KCl, 2 mM CaCl_2_, 1 mM MgCl_2_, and pH = 7.4) at 37°C and imaged 2 days later using Zeiss LSM700 confocal with a 63× water objective (NA: 1.4). Movies were captured using the 488 laser at 1.5%, a 3.5× optical zoom and a 256 × 256 pixel resolution for 250 cycles (1 second/cycle; Pathania et al., 2014). The pixel dwell time was set to 3.15 µsec and pinhole size was set to 2 µm. Bleaching of the GFP‐GLT‐1‐V5 with 100% 488‐laser intensity occurred after 10 cycles. Clustered and diffuse GFP‐GLT‐1‐V5 in astrocytes processes were selected for photobleaching. ImageJ was used for measuring the fluorescence intensity of a manually selected ROI normalized to the total fluorescence of the image to correct for photobleaching. These values were normalized to the mean of the 10 frames prior to bleaching, and the lowest value in the dataset was subtracted from all values. Finally, the recovery data points were fitted to an exponential recovery curve {*y* = a*[1 − exp(−b*x)]} using Mathematica (Wolfram Research) and the average time constant was calculated as *τ* = 1/*b* where *b* is the rate constant. The mobile fraction was calculated as an average of the plateaued fluorescence level, taken as the last 20 frames, and presented as a percentage of the prebleached level.

### Single Particle Tracking (SPT)

Fluorescence was captured using an Olympus microscope (BX51WI) with a 60× water objective (NA: 0.9) Olympus objective coupled to an EM‐CCD camera (Ixon; Andor; Eckel et al., 2015; Muir et al., 2010; Muir and Kittler, 2014; Smith et al., 2012). Excitation was provided by a mercury‐spiked xenon arc lamp (Cairn) or by a metal‐halide lamp (X‐Cite120, EXFO). Appropriate filters were chosen for GFP‐tagged constructs, Quantum Dots (QDs), and FM 4‐64. The imaging media used for all experiments contained 125 mM NaCl, 5 mM KCl, 1 mM MgCl_2_, 2 mM CaCl_2_, 10 mM d‐glucose, and 10 mM Hepes and was adjusted to pH = 7.4 with NaOH before use. Cells were imaged under perfusion (1.5 mL/min) and heating (35–37°C). For recovery experiments, l *‐*Glutamate (100 µM, Sigma) alone or with TFB‐TBOA (10 µM, Tocris) were applied 3 min in the perfusion during imaging. 4‐AP (1 mM, Sigma) and TTX (1 µM, Tocris) were applied 20 min before imaging.

Labeling of GFP‐GLT‐1‐V5 with QD on dissociated cultures was performed by first incubating the coverslips with the mouse anti‐V5 antibody (10 µg/mL, Invitrogen) at RT for 6 min, then incubating for 2 min with an antimouse 605‐nm QD (0.5 nM, Invitrogen) in imaging media containing 10% horse serum to block nonspecific binding.

For labeling of GFP‐GLT‐1‐V5 with QD on slice cultures, 2 µL of mouse anti‐V5 antibody and 1 µL of anti‐mouse 605‐nm QD were mixed in a total volume of 10 mL of imaging medium +1% BSA and vortexed for 5–10 min at RT and at the lowest speed. A 35‐mm Petri dish was covered with parafilm and a drop of 150 µL of the anti‐V5 QDs‐labeled diluted 1 in 1000 in imaging medium + 1% BSA where the slices with the membranes have been incubated for 10 min at 37ºC. Slices were washed 3 times in imaging medium and imaged thereafter. QD movies were recorded at 8.5 Hz. Labeling of active presynaptic terminals with FM 4‐64 (Invitrogen) was performed by a four‐step protocol in imaging media at RT: 10 µM FM 4‐64 and 50 mM KCl (1 min); 2 µM FM4‐64 (1 min); imaging media only (20 s); imaging media only (20 s).

### Multiwavelength Imaging and Analysis (Optosplit)

An inverted Zeiss Axiovert 200 microscope (63× 1.4 NA oil objective), attached to an Evolve (EMCCD) camera (Photometrics), fitted with an image splitter (Optosplit II, Cairn Research), allowed simultaneous acquisition of images at two separate emission wavelengths. Videos were recorded at 8.5 Hz using Micro‐manager software (Edelstein et al., 2010). Excitation was achieved through a D470/40× filter (Chroma) and emission was split using a 565DCXR dichroic beam‐splitter (Chroma), subsequently collecting with HQ522/40M and HQ607/75M (Cairn Research) filters for SyGCaMP5 and Qdot 605, respectively. A Grass S9 stimulator and a stimulation bath (Warner Instruments) allowed field stimulation (10 Hz for 10 s) of neuron‐astrocyte cocultures prepared as described previously. Movies were aligned using the Cairn Image Splitter plugin in ImageJ. Graphs showing Δ*F*/*F*_0_ were plotted using “Mathematica” (Wolfram Research). Regions of Interest were manually drawn and after background subtraction, fluorescence was normalized to the first 10 frames. QD were tracked automatically in ImageJ by using Mosaic particle tracker 2D/3D plugin (8), only QD tracks in the neighboring astrocytic processes with their midpoint within 8 pixels (<2 µm) of a SyGCaMP5 centroid before the field stimulation were selected and their coordinates were used to estimate instantaneous diffusion coefficients as described below.

### Image and Statistical Analysis

All experiments were performed on astrocytes from at least three individual preparations.

For analysis of intensity measurements in clusters, analysis was performed in ImageJ by the following method: The StackReg macro was used to correct for minor coverslip drift (Thevenaz et al., 1998) then regions were drawn that captured cluster positions across the image stack. Graphs showing cluster index (*V*/*V*_0_) were plotted using “Mathematica” (Wolfram Research). Regions of Interest were manually drawn. After background subtraction, standard deviation was normalized to the first 10 frames (50 s) of the movie (Cluster Index). Images were acquired every 5 s. The standard deviation from the consecutive frames ending at *t* = 15 min was used for analysis and comparison. *P* values given are from two‐tailed t tests. Values are given as mean SD; error bars represent SEM (standard error of the mean).

Automated QD detection and trajectory reconstruction was performed, as previously described (Muir and Kittler, 2014; Pathania et al., 2014). The mean squared displacement (MSD) versus time (*t*) was calculated for each QD track. Instantaneous diffusion coefficients (*D*) were then estimated by fitting a line to the first five points of the MSD curve, using the 2D diffusion law (MSD = 4Dt). QD track segments were classified as synaptic if their midpoint was within 0.5µm (2 pixels) of a FM 4‐64 centroid. Box plots display the interquartile range of diffusion scores, with the median score highlighted. Error bars represent the SEM. Non‐Gaussian data sets were tested by nonparametric Mann‐Whitney test. Indications of significance correspond to *P*‐values *P* < 0.05 (*), *P* < 0.01, (**) and *P* < 0.001 (***).

## Results

### GLT‐1 Clustering and Surface Diffusion in Astrocytes Cultured Alone

**Figure 1:**
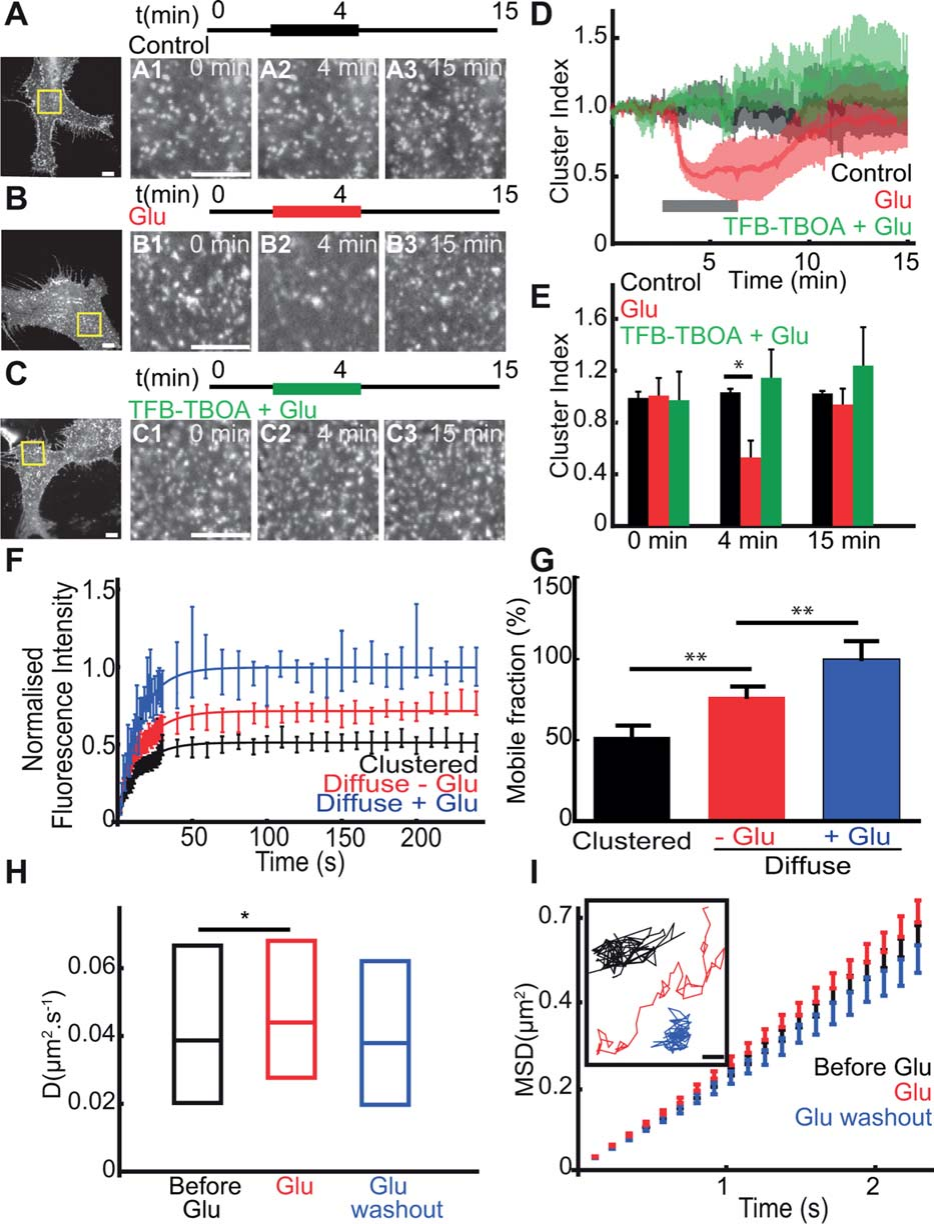
**Regulation of GLT‐1 surface diffusion by glutamate is transporter activity dependent.** Astrocytes cultured alone and transfected with GFP‐GLT‐1 or GFP‐GLT‐1‐V5 and imaged live after 4 to 7 days expression. **A**–**C**: Representative time lapse imaging illustrates the GFP‐GLT‐1 clusters in control untreated astrocytes (A), treated with 100 μM Glu (B) or with 10 μM TFB‐TBOA + 100 μM Glu (C). Control images before treatment of a whole astrocyte (left panel). Schematic of recovery experiments. Regions boxed in left panels shown before treatment at 0 min, during treatment at 4min and after recovery at 15 min. Scale bars, 10 μm. **D**: Time course of GFP‐GLT‐1 de‐clustering (cluster index, V/V0) in astrocytes control untreated (black, *n* = 7 cells), Glu treated (green, *n* = 8 cells) and Glu + TFB‐TBOA treated (red, *n* = 7 cells) astrocytes. **E**: Cluster index at 0, 4, and 15 min. Loss of fluorescence in GFP‐GLT‐1 clusters at 4 min is significant compared with untreated control (*P* = 0.023) but is not significantly different at 15 min (*P* > 0.05). **F**: Fluorescence Recovery after Photobleaching. Quantification of GFP fluorescence intensity shows the recovery of diffuse GLT‐1 (red) is greater than clustered GLT‐1 (black) and that glutamate increases GLT‐1 recovery (blue). Data points represent an average of movies and are fitted with single exponentials (black, red, and blue lines). **G**: The mobile fraction, quantified as the final amount of recovered fluorescence presented as a percentage of the total bleached fluorescence, for diffuse GLT‐1 (black, *n* = 12 videos, 74.67 ± 4.890) is significantly increased compared with that for GLT‐1 in clusters (red, *n* = 9 videos, 51.92 ± 5.120; *P* = 0.002, t test) and GLT‐1 in glutamate is significantly increased compared with before treatment (blue, *n* = 6 videos, 100.2 ± 8.769; *P* = 0.01, t test). **H**: Instantaneous diffusion coefficients and (**I**) MSD versus time, MSDt plot, and representative single trajectories, Scale bar, 0.5 μm of QD‐tagged GFP‐GLT‐1‐V5. Control before treatment (black, median = 0.039 μm^2^/s; *n* = 579 trajectories), after 2 min with 100 μM glutamate (red, median = 0.044 μm^2^/s; *n* = 504 trajectories) and after drug washout (blue, median = 0.038 μm^2^/s; *n* = 276 trajectories). Median D is significantly increased upon 100 μM glutamate treatment (*P* = 0.005, Mann‐Whitney test) but is not significantly different after glutamate washout (*P* > 0.05).

As astrocytic GLT‐1 is the major astrocytic transporter involved in glutamate clearance, we first determined the effect of glutamate application on GLT‐1 surface diffusion in the absence of neuronal signalling. To achieve this, we expressed GLT‐1 with an N‐terminal GFP tag (GFP‐GLT‐1) in primary astrocyte cultures and investigated GLT‐1 surface clustering and diffusion dynamics on the astrocyte surface using live‐cell imaging of the GFP fluorescence. We found that under basal conditions, GFP‐GLT‐1 formed bright fluorescent clusters in the astrocyte soma and along processes (Fig. 1A–C). Continuous imaging revealed that the intensities and locations of GFP‐GLT‐1 clusters were stable for periods of 15 min (Fig. 1A). Interestingly, GLT‐1 clusters rapidly and reversibly dispersed during acute glutamate application (100 µM, 3 min, Fig. 1B). The glutamate‐induced loss of GFP‐GLT‐1 clustering was marked by a 50% decrease in the clustering index followed by a complete recovery after washout (Fig. 1D,E). Next, to examine the influence of transporter activity on GLT‐1 clustering, we investigated the effect of a GLT‐1 transporter inhibitor on glutamate induced cluster dispersal. Intriguingly, blocking transporter activity with TFB‐TBOA, a non‐transportable competitive inhibitor prevented the glutamate induced GLT‐1 cluster dispersal (Fig. 1C–E). We further explored the surface mobility of clustered GFP‐GLT‐1 transporters compared to diffuse GFP‐GLT‐1 using FRAP (Supp. Info. Fig. S1). Interestingly, the fluorescence recovery of diffuse GLT‐1 was higher in comparison to clustered GLT‐1 transporters, suggesting that GLT‐1 clustering increases GLT‐1 stability (Fig. 1F). This difference in GLT‐1 mobility was confirmed by a total mobile fraction of GFP‐GLT‐1 of 52% in clustered GFP‐GLT‐1 compared with 77% for the diffuse GFP‐GLT‐1 (*P* = 0.002, Fig. 1G). Furthermore, glutamate treatment (100 µM, 3 min) increased GLT‐1 fluorescence recovery and the mobile fraction of the diffuse GLT‐1 to 100% compared to control, before treatment (*P* = 0.008, Fig. 1G). Thus, clustered GLT‐1 transporters are less mobile than diffuse GLT‐1 transporters, and GLT‐1 cluster dispersal upon glutamate treatment correlates with increased GLT‐1 mobility. Next, we investigated the lateral mobility of GLT‐1 molecules on the astrocyte surface in response to glutamate treatment by using SPT with QDs. For SPT experiments we used a version of GFP‐GLT‐1 with a V5 extracellular tag inserted in the large GLT‐1 extracellular loop (Supp. Info. Fig. S2A). By QD tracking we found that GFP‐GLT‐1‐V5 lateral mobility was significantly increased in response to glutamate (100 µM, 3 min), which was reversed upon glutamate washout (Fig. 1H). Moreover, the GLT‐1 single trajectories explored a greater area upon glutamate treatment, correlating with increased GLT‐1 lateral diffusion (Fig. 1H). Interestingly, despite the formation of stable GLT‐1 clusters, we found a linear MSDt plot, reflecting free diffusion (Brownian motion) of GLT‐1 molecules rather than a confined behavior (Fig. 1I) suggesting a large proportion of GLT‐1 is found outside clusters.

Together, these data reveal that in cultured astrocytes GLT‐1 can form stable clusters, while individual GLT‐1 molecules can be highly mobile on the astrocyte surface. Glutamate application leads to GLT‐1 cluster dispersal that is dependent on GLT‐1 transporter activity, which correlates with an increase in GLT‐1 surface diffusion in the astrocyte membrane.

### GLT‐1 Is More Stable and More Confined inside Synaptic Areas under Basal Conditions

**Figure 2:**
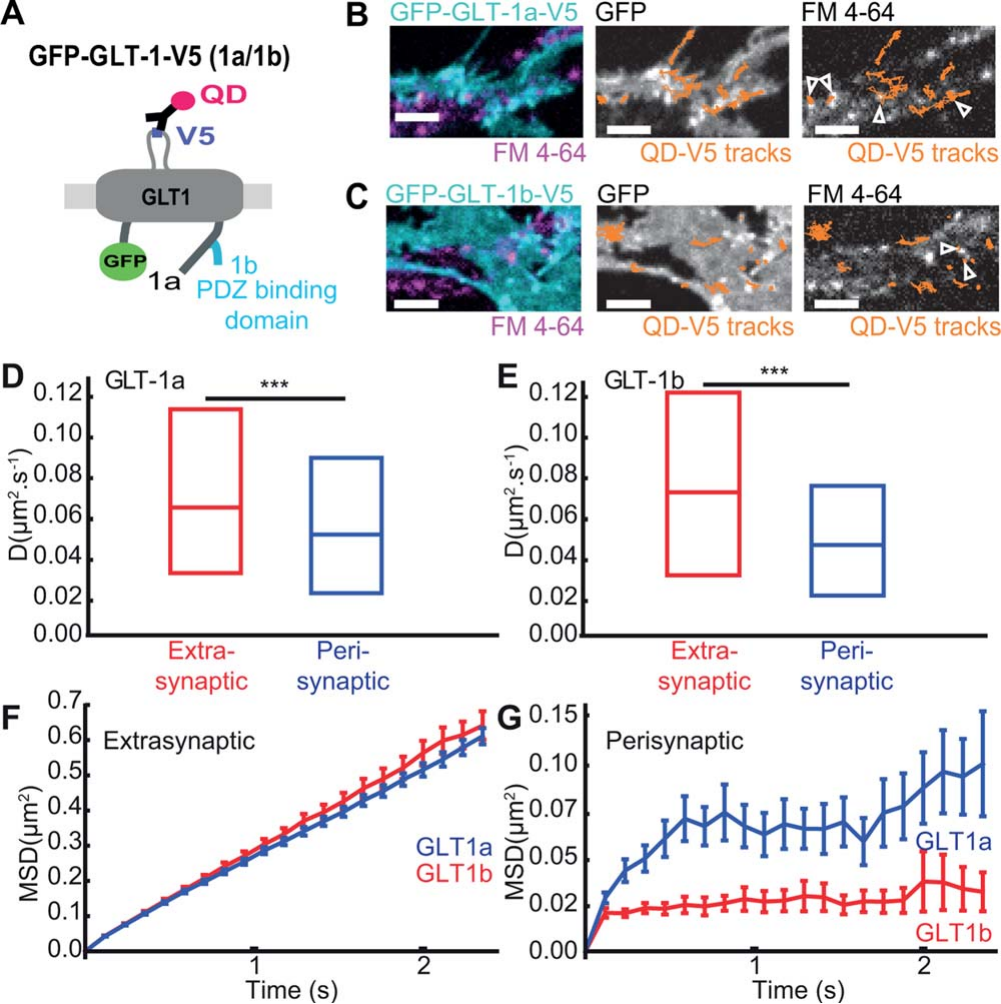
**GLT‐1 is stable and confined inside synaptic areas under basal conditions.** Astrocytes in hippocampal neuron‐astrocyte mixed culture transfected with GFP‐GLT‐1a‐V5 or GFP‐GLT‐1b‐V5 at DIV10 and imaged at DIV13 after FM4‐64 staining. **A**: Schematic representation of GFP‐GLT‐1(a/b)‐V5 labelled by an anti‐V5 antibody/QD complex. **B,C**: Representative time lapse imaging illustrates GFP‐GLT‐1a‐V5 (B) and GFP‐GLT‐1b‐V5 (C) in a region of an astrocyte. GFP‐GLT‐1‐V5 overlaid with FM4‐64 stained synapses (left panels), or by QD‐tagged GFP‐GLT‐1‐V5 trajectories shown in orange (middle panels), and FM4‐64 stained synapses overlaid by QD‐tagged GFP‐GLT‐1‐V5 trajectories shown in orange (right panels), Scale bars 5 μm. **D**: Instantaneous diffusion coefficients of extrasynaptic GLT‐1a (red, median = 0.065 μm^2^/s; *n* = 599 trajectories) and perisynaptic GLT‐1a (blue, median = 0.052 μm^2^/s; *n* = 112 trajectories), median D is significantly decreased in perisynaptic areas (P= 6x10 ^− 3^, Mann‐Whitney test). **E**: Instantaneous diffusion coefficients of extrasynaptic GLT‐1b (red, median = 0.071 μm^2^/s; *n* = 255 trajectories) and perisynaptic GLT‐1b (blue, median = 0.047 μm^2^/s; *n* = 62 trajectories). Median D is significantly decreased in perisynaptic areas (*P* = 8 × 10^−5^, Mann‐Whitney test). MSDt plot of extrasynaptic (**F**) and perisynaptic (**G**) GLT‐1a and GLT‐1b.

Given the glutamate induced alteration in GLT‐1 surface diffusion, next we wanted to determine whether the presence of neurons and synaptic contacts could affect astrocytic GLT‐1 surface dynamics. Interestingly, we observed that in the presence of neurons there was a 2 fold increase in GLT‐1 surface diffusion in astrocyte‐neuron cocultures in comparison to GLT‐1 in astrocytes cultured alone (Supp. Info. Fig. S2B), revealed by more mobile transporters on the astrocyte surface in presence of neurons (Supp. Info. Fig. S2C and S2D) as also recently reported by Murphy‐Royal. GLT‐1a and GLT‐1b are the two main isoforms of GLT‐1 expressed in forebrain and differ only in their intracellular C‐terminal sequence (Fig. 2A). Unlike GLT‐1a, GLT‐1b has a PDZ binding domain that interacts with scaffolding proteins (Bassan et al., 2008; Gonzalez‐Gonzalez et al., 2008a; Underhill et al., 2015). Hence, we next wanted to determine whether astrocytic GLT‐1a and GLT‐1b transporters exhibited differential surface diffusion properties, depending on their proximity to active synapses. We expressed GFP‐GLT‐1a‐V5 (Fig. 2B) or GFP‐GLT‐1b‐V5 (Fig. 2C) in hippocampal astrocyte‐neuron coculture. SPT using QDs along with simultaneous labeling of the active presynaptic terminals using FM4‐64 (Supp. Info. Fig. S3A), allowed us to track the GLT‐1 transporters in regions of the astrocyte plasma membrane located in close proximity to synapses (hence forth termed perisynaptic locations) or in astrocyte regions that were not in proximity to synapses (termed extrasynaptic locations) (Fig. 2B and Supp. Info. Fig. S2C).

Interestingly, the median lateral diffusion of perisynaptic GLT‐1a, *Dperi* was 0.052 µm^2^/s, whereas the extrasynaptic GLT‐1a, *Dext* was 0.065 µm^2^/s, and for GLT‐1b, *Dperi* was 0.047 µm^2^/s, whereas the extrasynaptic GLT‐1b, *Dext* was 0.071 µm^2^/s (Fig. 2D,E). This reveals that astrocytic surface diffusion of extrasynaptic GLT‐1 is more dynamic than perisynaptic GLT‐1 for both isoforms. No significant difference was observed between GLT‐1a and GLT‐1b median lateral diffusion. Interestingly, MSDt plot of GLT‐1a and GLT‐1b revealed differential properties depending on their localisation relative to the synapse (Fig. 2F,G). The MSDt plot of extrasynaptic GLT‐1a and GLT‐1b exhibited linear profiles, indicating Brownian diffusion, however, perisynaptic GLT‐1a and GLT‐1b exhibited sublinear curvature, indicative of confined motion. In addition, the differences in transporter mobility were due to a shift of the diffusive fraction without a significant change of the immobile fraction (data not shown). Furthermore, comparison of the extrasynaptic GLT‐1a and GLT‐1b MSDt plots revealed no significant difference (Fig. 2F). However, comparison of the perisynaptic GLT‐1a and GLT‐1b MSDt plots revealed that GLT‐1b was significantly more confined compared to GLT‐1a (Fig. 2G) and the residency time of GLT‐1b within the perisynaptic area is significantly longer than GLT‐1a (Supp. Info. Fig. S3B).

Together, these data showed that for both isoforms, perisynaptic GLT‐1 is more stable than GLT‐1 in extrasynaptic regions under basal conditions. Moreover, perisynaptic GLT‐1b is more stable than perisynaptic GLT‐1a suggesting a role of GLT‐1b PDZ interactors in its confinement.

### Glutamate and Neuronal Activity Regulate the Surface Diffusion of GLT‐1 in Astrocytes

Next, we were interested in further studying the effect of neuronal activity on GLT‐1 surface dynamics in astrocytes. As GLT‐1a and GLT‐1b showed similar behavior in response to the pharmacological treatments; here, we have shown the results of GLT‐1a isoform only. For the following experiments, we expressed GFP‐GLT‐1a‐V5 in hippocampal astrocyte‐neuron coculture and performed single‐particle tracking experiments.

**Figure 3:**
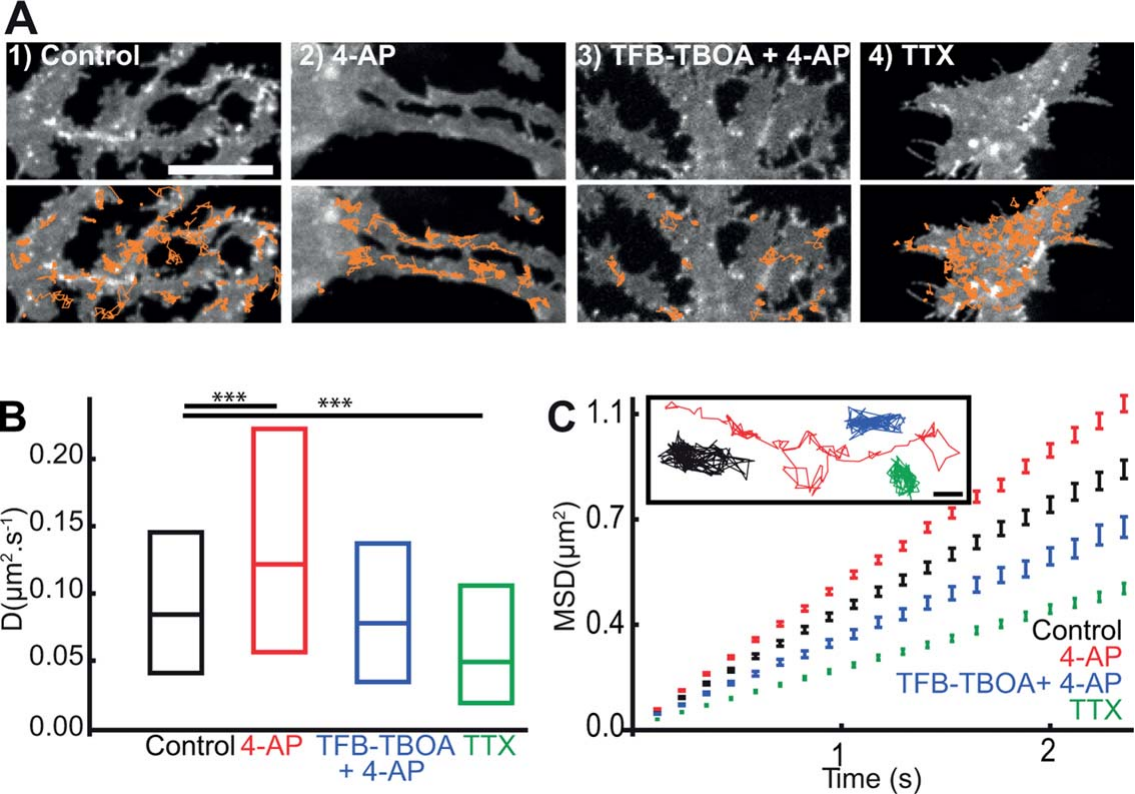
**Neuronal activity mediated GLT‐1 surface diffusion increase is transporter activity dependent**. Hippocampal neuron‐astrocytes mixed culture transfected with GFP‐GLT‐1a‐V5 at DIV10 and imaged at DIV13. **A**: Representative time lapse imaging illustrates GFP‐GLT‐1a‐V5 in a region of interest of an astrocytic process (top panel) and overlaid by QD‐tagged GFP‐GLT‐1a‐V5 trajectories shown in orange (bottom panels) in control untreated (1), treated 20 min with 1 mM 4‐AP (2), 20 min with TFB‐TBOA 10 μM + 4‐AP 1mM (3) or 1 µM of TTX (4), Scales bars 10 μm. Instantaneous diffusion coefficients (**B**) and MSDt plot and single trajectories (**C**) of QD‐tagged GFP‐GLT‐1‐V5. Control untreated (black, median = 0.08 μm^2^/s; *n* = 1061 trajectories), after 20 min with 1 mM 4‐AP (red, median = 0.12 μm^2^/s; *n* = 779 trajectories), after 20 min of TFB‐TBOA 10 μM + 4‐AP 1 mM (blue, median = 0.08 μm^2^/s; *n* = 282 trajectories) and after 20 min with 1 µM of TTX (green, median = 0.05 μm^2^/s; *n* = 1191 trajectories). Median D is significantly increased upon 4‐AP (*P* = 2 × 10^−14^, Mann‐Whitney test) and is significantly decreased upon TTX (*P* = 1.2 × 10^−14^, Mann‐Whitney test) but is not significantly different upon TFB‐TBOA 10 μM + 4‐AP 1 mM (*P* > 0.05).

Similarly to experiments in astrocytes cultured alone (Fig. 1H,I), we noticed a rapid and reversible loss of clustered GFP‐GLT‐1a‐V5 fluorescence (Supp. Info. Fig. S4A) and an increase in GLT‐1a surface diffusion upon glutamate treatment, which are prevented when the transport activity of astrocytic glutamate transporters is inhibited with TFB‐TBOA (Supp. Info. Fig. S4B and S4C). Since glutamate is released during neuronal firing, we studied the effect of increased neuronal activity on the GLT‐1a surface dynamics via 4‐AP (a nonselective voltage‐dependent K+‐channel blocker) treatment in astrocyte‐neuron cocultures (Fig. 3). Similar to glutamate treatment, we observed a loss of clustered GFP‐GLT‐1a‐V5 fluorescence upon 4‐AP (1 mM, 20 min), which is prevented in the presence of the transporter inhibitor (Fig. 3A1–3). We found significantly increased GLT‐1a (71%, Fig. 3B) surface diffusion following 4‐AP treatment, characterized by more mobile transporters (Fig. 3C). Furthermore, inhibition of GLT‐1 transporter activity with TFB‐TBOA blocked the 4‐AP dependent increase in GLT‐1a lateral diffusion (Fig. 3B), reverting it to control levels. The MSDt plot exhibited an upward linear shift, indicative of Brownian motion and increased lateral diffusion upon 4‐AP treatment (Fig. 3C). These data suggest that stimulating neuronal activity triggers an increase in GLT‐1 surface mobility probably due to the increased glutamate release upon 4‐AP treatment (Girault et al., 1986).

In contrast, inhibiting neuronal firing with TTX (a highly selective sodium channel blocker) treatment, led to an increase in clustered GFP‐GLT‐1a‐V5 (Fig. 3A4) and significantly decreased GLT‐1a surface diffusion (35%, Fig. 3B), correlating with less mobile transporters (Fig. 3C). The MSDt plot exhibited a downward linear shift, indicative of decreased Brownian motion, but not confinement upon TTX treatment (Fig. 3C). This result confirmed that inhibition of neuronal activity decreased GLT‐1 surface diffusion (Murphy‐Royal et al., 2015). Glutamate and neuronal activity can also regulate the surface diffusion dynamics of GLT‐1b (Supp. Info. Figs. S5 and S6). Since the majority of the GLT‐1 trajectories in the mixed cultures were from the extrasynaptic GLT‐1 population (87 and 80% of the total GLT‐1a and GLT‐1b trajectories, respectively), we propose that pharmacological alterations in neuronal activity can influence surface diffusion of the extrasynaptic GLT‐1 transporters. Together, these data showed that neuronal activity bidirectionally regulates GLT‐1a and GLT‐1b surface mobility on the surface of astrocytes in a GLT‐1 transporter activity‐dependent manner.

**Figure 4:**
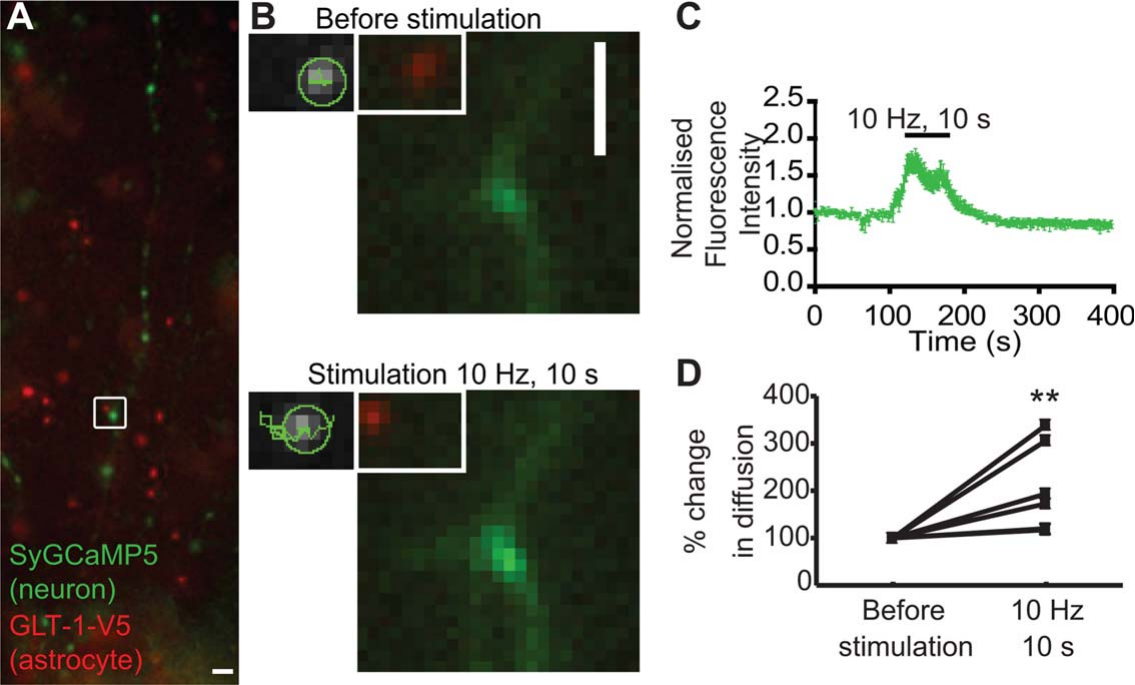
**Electrical stimulation mediates GLT‐1 transporter surface mobility at activated synapses. A**: Representative images of hippocampal neurons transfected with SyGCaMP5 (green) at 7‐10 DIV, cocultured with astrocytes pretransfected with GLT‐1‐V5 (Red). Cocultures were maintained for 3‐4 DIV and imaged using an image splitter. Scale bar, 10 μm. **B**: Region boxed in (A) and representative single trajectories shown before stimulation (top panel), and during (bottom panel, 10 Hz for 10 s, 100 PA). Scale bar, 2 μm. **C**: Time course of SyGCaMP5 fluorescence F/F0 (6 synapses). Background signal at each corresponding time point was subtracted from the SyGCaMP5 signal before normalizing to the first 10 frames (F/F0). **D**: Percentage of change in GLT‐1 surface diffusion significantly increased during stimulation compared to before (*n* = 6 trajectories, *P* = 0.04, Mann‐Whitney test).

Next, to determine the effect of neuronal activity on GLT‐1 surface diffusion in astrocytic processes in apposition to synapses undergoing activation, we performed a more physiological form of neuronal stimulation (electrical field stimulation) of hippocampal neurons transfected with synaptically targeted GCaMP5 (SyGCaMP5) to label stimulated synapses and co‐cultured with astrocytes pre‐transfected with GLT‐1‐V5 (Fig. 4A). By using an image splitter, we were able to simultaneously image the neuronal SyGCaMP5 signal and QD‐labelled GLT‐1 on the astrocyte surface (Fig. 4B). Upon field stimulation (10 Hz for 10 s, 100 APs), a 2‐fold increase was observed in SyGCaMP5 signal, showing that field stimulation drives increased presynaptic calcium signal and revealing an increase in synaptic activity and position of activated synapses (Fig. 4C). Interestingly, we observed that field stimulation increased GLT‐1 surface diffusion in apposition to activated synapses by 100% compared with before stimulation (Fig. 4D). The activity‐dependent increase in GLT‐1 surface diffusion is likely due to the glutamate released in the synaptic sites during the stimulation.

### Glutamate and Neuronal Activity Regulate GLT‐1 Surface Diffusion in *Ex‐Vivo* Brain Slices

**Figure 5:**
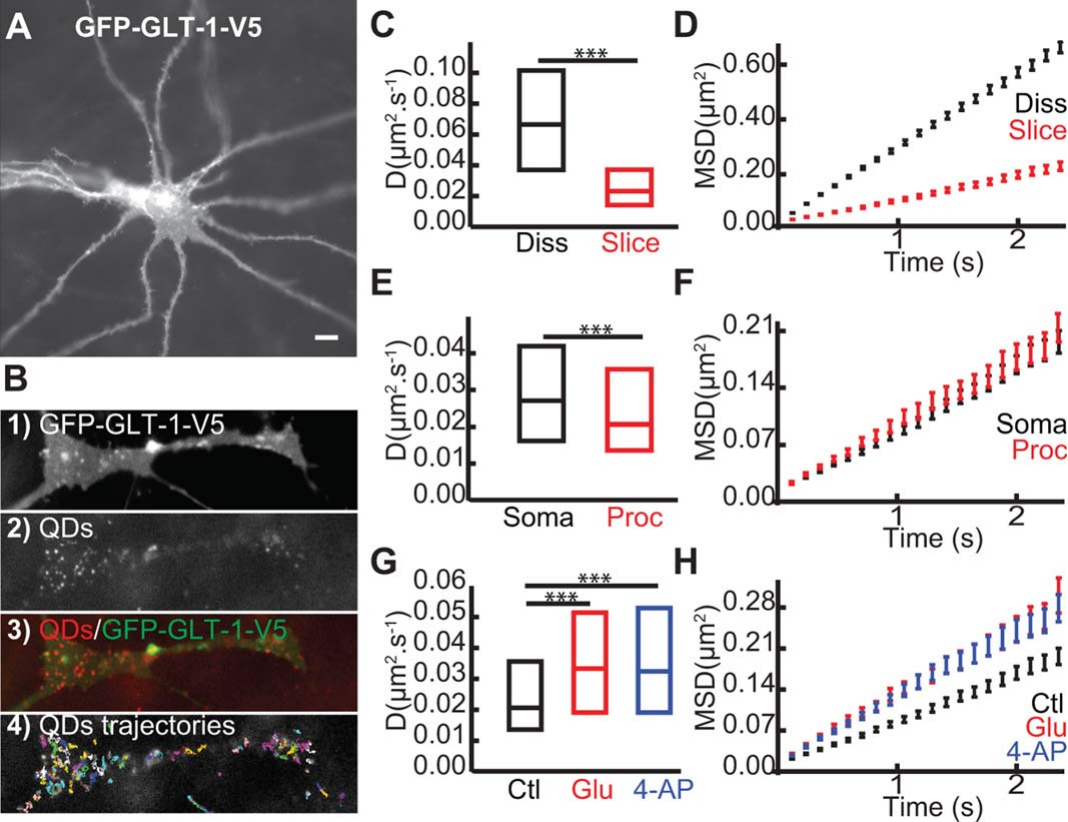
**Glutamate and neuronal activity regulate GLT‐1 surface diffusion in brain slices.** A: Example of astrocyte expressing GFP‐GLT‐1‐V5 and imaged 3–5 days after transfection. Scale bar, 10 μm. **B**: Zoomed region on an astrocytic process expressing GFP‐GLT‐1‐V5 (B1), QDs (B2), QDs overlaid with GFP‐GLT‐1‐V5 (B3) and representative QD trajectories (B4). Instantaneous diffusion coefficients (**C**,**E**,**G**) and MSDt plot of QD‐tagged GLT‐1 (**D**,**F**,**H**). C,D: In dissociated culture (black, median *D* = 0.067 μm^2^/s; *n* = 783 trajectories) and in slice cultures (red, median = 0.021 μm^2^/s; *n* = 325 trajectories), median *D* is significantly decreased in slice cultures compared with in dissociated cultures (*P* = 3.5 × 10^−80^, Mann‐Whitney test). D,E: In soma (black, median *D* = 0.027 μm^2^/s; *n* = 108 trajectories) and in processes (red, median *D* = 0.021 μm^2^/s; *n* = 325 trajectories), median *D* is significantly decreased in processes compared with in soma (*P* = 0.005, Mann‐Whitney test). **G,H**: After 2 min of glutamate 100 μM (red, median *D* = 0.033 μm^2^/s; *n* = 234 trajectories), and after 20 min of 4‐AP 1 mM (blue, median *D* = 0.032 μm^2^/s; *n* = 174 trajectories), median *D* is significantly increased in both treatment conditions compared with control (*P*_glu_ = 1.15 × 10^−10^ and *P*_4‐AP_ = 0.03, Mann‐Whitney test).

Very little is known about GLT‐1 dynamics in more physiological and spatially complex tissue, like brain slices. To address this, we used hippocampal organotypic brain slices transfected with GFP‐GLT‐1‐V5 (Fig. 5A). In transfected astrocytes, GLT‐1 was expressed on the surface of the astrocytes and formed obvious clusters (Fig. 5A), corroborating previous data (Benediktsson et al., 2012). After incubating the slices with anti‐V5‐QD complexes, we were able to observe QD labeling throughout the slice, specific only to the transfected astrocytes (Fig. 5B1–4). First, we found a significant decrease in GLT‐1 surface diffusion in brain slices (slice) compared with in dissociated (*diss*) cultures (Fig. 5C, D*slice* was 0.021 µm^2^/s and D*diss* was 0.067 µm^2^/s) where the transporters explored a greater area (Fig. 5D). Moreover, despite a similar explored area by GLT‐1 in soma compared to processes (Fig. 5E) that could be due to more compactly packed cells and extracellular matrix, we found that GLT‐1 coefficient diffusion was significantly lower in processes (*proc*) compared to soma (Fig. 5E, D*proc* was at 0.021 µm^2^/s and D*soma* was 0.027 µm^2^/s). This reduction could be due to the presence on astrocytic processes of more synaptic sites, where we have previously shown that GLT‐1 is more confined (Fig. 2G). This data suggests that GLT‐1 is more stable in astrocytic processes in apposition to synapses in slice cultures under basal conditions. We then investigated the effects of glutamate (100 µM, 2 min) and stimulation of neuronal activity with 4‐AP (1 mM, 20 min) on GLT‐1 surface dynamics in astrocytic processes (Fig. 5G). Interestingly, surface GLT‐1 is more dynamic in brain slices when treated with glutamate (D*glu* = 0.033 µm^2^/s) or 4‐AP (D*4‐AP =* 0.032 µm^2^/s), in comparison to untreated slice control (D*control =* 0.021 µm^2^/s). Moreover, the GLT‐1 single trajectories explored a greater area upon glutamate and 4‐AP treatment, correlating with increased GLT‐1 lateral diffusion (Fig. 5H). In addition to validating our results obtained from dissociated cultures that showed neuronal activity mediated regulation of GLT‐1 surface dynamics, these data are evidence that GLT‐1 modulates its surface diffusion upon stimulation of neuronal activity in brain slices.

## Discussion

GLT‐1 glutamate transporters are critical modulators of extracellular glutamate in the brain (Danbolt, 2001; Tzingounis and Wadiche, 2007) and regulating their surface diffusion and clustering properties to modulate transporter number at synaptic release sites could be a rapid mechanism for locally tailoring glutamate uptake to shape glutamatergic neurotransmission. Here, by using GFP‐time lapse imaging, FRAP and single‐particle tracking with QD, we report the behavior of the two main GLT‐1 transporter isoforms (GLT‐1a and GLT‐1b) in astrocytic surface domains, under both basal conditions and in response to neuronal activity. GLT‐1a and GLT‐1b, were both found to be highly dynamic at non‐synaptic locations and to exhibit a more confined diffusion when present in astrocyte membranes adjacent to synapses, with GLT‐1b more so. Moreover, exogenous glutamate application or alteration of neuronal firing led to altered GLT‐1 surface diffusion in a transporter activity dependent manner and to an increase in GLT‐1 mobility in proximity to activated synapses. Importantly, we could also observe for the first time neuronal activity‐dependent alterations in GLT1 surface mobilities in astrocyte processes *in situ* in brain slices.

In cultured astrocytes GLT‐1 transporters could form stable clusters that were rapidly and reversibly dispersed upon glutamate treatment. Interestingly, the glutamate‐dependent increase in GLT‐1 de‐clustering and surface diffusion was blocked by the nontransportable competitive inhibitor, TFB‐TBOA, suggesting a critical role for the transport activity of GLT‐1 in regulating its surface dynamics. Indeed, application of aspartate, another glutamate transporter substrate also led to GLT‐1 cluster dispersal (data not shown), reinforcing the idea that substrate binding to the GLT‐1 transporter can directly affect its surface diffusion. In agreement with this, inhibition of GLT‐1 transport activity with TBOA under basal conditions decreased steady state GLT‐1 surface diffusion in mixed neuron‐glial cultures (Murphy‐Royal et al., 2015) presumably by inhibiting GLT‐1 activation by endogenous glutamate in the cultures. GLT‐1 is mainly found in oligomeric form on the astrocyte surface (Haugeto et al., 1996), which may facilitate the formation of the GLT‐1 clusters observed in our study, although FRAP and single‐particle tracking resolution do not allow us to distinguish between the different surface oligomeric GLT‐1 forms. GLT‐1 can also interact with scaffolds (Bassan et al., 2008; Gonzalez‐Gonzalez et al., 2008a; Underhill et al., 2015), which could also contribute to cluster formation. Substrate‐mediated GLT‐1 cluster dispersal could be due to the change in GLT‐1 conformation demonstrated in the presence of its substrates (Qu and Kanner, 2008; Yernool et al., 2004), which could facilitate oligomer disassembly or uncoupling from intracellular scaffolds.

Although a recent study reported the surface diffusion dynamics of the GLT‐1a isoform (Murphy‐Royal et al, 2015), the activity‐dependent behavior of GLT‐1b was not known. On studying the two isoforms, we identified two populations for both GLT‐1 isoforms based on their basal surface mobilities in the astrocyte membrane: a perisynaptic population, characterized by a slower surface diffusion and a more mobile extrasynaptic population. This is similar to the confinement of neurotransmitter receptors observed at postsynaptic sites (Choquet and Triller, 2013), promoting the importance of specialized astrocytic subdomains proximal to synapses. The two predominant astrocytic GLT‐1 isoforms, GLT‐1a and GLT‐1b, differ only in their extreme intracellular C‐terminal sequences. Unlike GLT‐1a, GLT‐1b, has a PDZ binding domain that binds to PDZ containing proteins such as PICK1, PSD95, and DLG1 (Bassan et al., 2008; Gonzalez‐Gonzalez et al., 2008a; Underhill et al., 2015). The confined motion seen for both perisynaptic GLT‐1a and GLT‐1b may be due to protein interacting motifs located in the N‐terminal or C‐terminal regions common to both isoforms. Interestingly, we observed that GLT‐1b was more confined in astrocyte domains close to synapses than GLT‐1a under basal conditions, supporting the concept that the GLT‐1b C‐terminal PDZ domain interacts with scaffolding proteins, anchoring it to macromolecular glial complexes located in subcellular domains opposite to neuronal presynaptic sites. Furthermore, the interaction between the PICK1 scaffolding protein and GLT‐1b has functional consequence on glutamate transport activity (Sogaard et al., 2013). GLT‐1a is known to form heteromers with GLT‐1b (Haugeto et al., 1996; Peacey et al., 2009) and since GLT‐1b interacts with multiple scaffolding proteins (Gonzalez‐Gonzalez et al., 2009; Underhill et al., 2015), it could serve to stabilize perisynaptic GLT‐1a, under basal conditions.

We also explored the activity‐dependence of GLT‐1 synaptic localisation to neuronal activity. MNI‐glutamate uncaging experiments on cultured astrocytes provided an initial suggestion that a glutamate rise could act locally to regulate GLT‐1 surface mobility (Murphy‐Royal et al., 2015). However, in those experiments putative synapses were labeled by staining for mitochondria which also densely populate dendrites and astrocyte processes themselves (Jackson and Robinson, 2015; Stephen et al., 2015) and therefore cannot equivocally label synaptic sites. To directly assess the local effect of physiologically released glutamate on GLT‐1 surface diffusion, we evoked action potentials to trigger synaptic glutamate release while directly visualizing active synapses using SyGCaMP5 fluorescence imaging. This, for the first time, revealed that an increase in GLT‐1 surface diffusion in the neighboring astrocyte processes surrounding the activated synapse. We speculate that during neuronal activity, synaptically released glutamate is taken up by GLT‐1 transporter, resulting in a change in GLT‐1 conformation, which could lead to the dissociation of GLT‐1 from its scaffolding proteins to allow for increased surface mobility. It will be interesting in the future to determine the scaffold protein(s) responsible for stabilizing GLT‐1 in astrocyte processes close to synapses but it is tempting to speculate that DLG1 or PICK1 may play a role.

To extend our findings to a more physiological setting, we used organotypic rat hippocampal brain slices, which exhibit functional local synaptic circuitry and preserved brain architecture. Interestingly, GLT‐1 transporters exhibited a more confined mobility in slice cultures in comparison to dissociated cultures. This could be due to the more compactly packed cells and extracellular matrix, and increased number of neuron‐glial contacts or due to increased expression levels of scaffold proteins within astrocytes in slices, which could restrict GLT‐1 mobility. Moreover, GLT‐1 transporters were more confined in astrocyte processes compared to the cell soma in organotypic slices, possibly due to a high coverage of synaptic sites on astrocytic processes *in situ* (Ventura and Harris, 1999). Importantly, although GLT‐1 was more stable in astrocyte processes in *ex vivo* brain slices (compared with dissociated culture), exogenous glutamate application or pharmacological activation of neuronal activity nonetheless led to a dramatic increase in GLT‐1 surface diffusion, providing compelling evidence that the activity dependent alteration of GLT‐1 mobility is an important mechanism for regulating GLT‐1 distribution in astrocytes in intact networks.

Although the transport cycle of GLT‐1 is slow (12–70 ms/cycle) in comparison to the duration of glutamate in the synaptic cleft (Bergles and Jahr, 1998; Clements et al., 1992; Wadiche et al., 1995), efficient glutamate clearance is facilitated by the presence of a large number of GLT‐1 transporters on the astrocyte surface, which can also buffer glutamate by binding it (Wadiche et al., 1995). In rat hippocampal slices, it has been shown that perisynaptic astrocyte processes (PAPs) are present at ∼62% synapse, with preference towards larger synapses that contain post synaptic densities (Witcher et al., 2007). PAPs express glutamate transporters (Derouiche et al., 2012; Pannasch et al., 2014) and are highly motile processes that are regulated by synaptically released glutamate. PAPs selectively modify their coverage of dendritic spines around potentiated synapses, which in turn is associated with an increased stability of synapses (Bernardinelli et al., 2014). While changes in surface protein levels, by processes such as endocytosis and exocytosis will occur at longer time scale of minutes to hours, rapid glutamate‐dependent alterations in GLT‐1 surface diffusion may act as a complementary pathway for the regulation of transporter numbers and positioning at synapses. Our data and that of others (Murphy‐Royal et al., 2015), supports the hypothesis that rapid dispersal of glutamate‐bound GLT‐1 in regions of high extracellular glutamate (such as in proximity to activated synapses) could potentially leave space for positioning of unbound GLT‐1 to allow for efficient and selective glutamate buffering. Rapid displacement of glutamate‐bound GLT‐1 molecules to allow their exchange with unbound ones, may be particularly important in PAPs where the small size of the PAPs may limit transporter availability in close proximity to synapses. Rapid increases in GLT‐1 surface mobility may also contribute to limit glutamate spillover (Asztely et al., 1997) and, therefore, prevent neuronal excitotoxicity. Our data provides further support for the active role of astrocytes in regulating glutamatergic signaling and shed light on transporter surface diffusion as an additional rapid regulatory mechanism for modulating number of transporters at synaptic release sites to regulate glutamate clearance in the CNS.

## Supporting information

Supporting Information Figure 1

Supporting Information Figure 2

Supporting Information Figure 3

Supporting Information Figure 4

Supporting Information Figure 5

Supporting Information Figure 6

Supporting Information
