# Supplementary material for: Neuronal activity mediated regulation of glutamate transporter GLT‐1 surface diffusion in rat astrocytes in dissociated and slice cultures
Source: Glia. 2016 May 17;64(7):1252–64. doi: 10.1002/glia.22997 (PMC4915597; doi:10.1002/glia.22997)
Supplement: Supplementary file 7 — Supporting Information [file GLIA-64-1252-s007.docx]

**Supplementary Figure 1: Clustered GLT-1 are more stable than diffuse GLT-1 and glutamate increases GLT-1 diffusion.**

Astrocytes transfected with GFP-GLT-1-V5 and subjected to Fluorescence Recovery after Photobleaching (FRAP) live imaging 3-4 days later.  Astrocytes were imaged for 250 s and bleaching of the GFP-GLT-1-V5 occurred after the first 10 s. Representative images over time in seconds of clustered *(A)* and diffuse *(B,C)* GFP-GLT-1-V5 fluorescence recovery before *(B)* and after glutamate *(C)* treatment. The white squares highlight the bleached regions. Scale bar, 5 μm.

**Supplementary Figure 2: Glutamate mediates rapid and reversible GLT-1 surface diffusion increase.**

*(A)* Schematic representation of GFP-GLT-1-V5 labelled by an anti-V5 antibody/QD complex. Astrocytes transfected with GFP-GLT-1-V5 and imaged in live after 4 to 7 days expression. *(B)* Representative time lapse imaging illustrates the GFP-GLT-1-V5 clusters in a whole astrocyte cultured without neuron (top left panel) and overlaid by QD-tagged GFP-GLT-1-V5 trajectories shown in orange (top right panel), or with neurons (bottom left panel) and overlaid by QD-tagged GFP-GLT-1-V5 trajectories shown in orange (bottom right panel), scale bar: 10μm. *(C)* Instantaneous QD-tagged GFP-GLT-1-V5 diffusion coefficients in astrocytes cultured without neurons (red), median = 0.039μm^2^/s; n = 2234 trajectories or with neurons (blue) median = 0.078μm^2^/s; n = 3221 trajectories (P=3.25x10^-112^, Mann-Whitney test). *(D)* Representative single trajectory of QD-tagged GFP-GLT-1-V5 surface diffusion in an astrocyte neuron-free culture (red) or with neuron (blue), scale bar: 0.5μm. Mean squared displacement versus time for QD-tagged GFP-GLT-1-V5. MSDt exhibit linear shape indicating free diffusion behaviour. Note the higher degree of QD-tagged GFP-GLT-1-V5 diffusion with neurons compared to without.

**Supplementary Figure 3: GLT-1b spends more time at synaptic sites than GLT-1a.**

*(A)* Representative time lapse imaging illustrates GFP-GLT-1a-V5 in an astrocyte (top left panel), QD-tagged GFP-GLT-1a-V5 (top right panel), FM4-64 staining synapses (bottom left panel) and GFP-GLT-1a-V5 overlaid by FM4-64 stained synapses (bottom right panels), Scale bars 20 μm. *(B)* Mean residency time of QD-tagged GFP-GLT-1a-V5 (GLT-1a, 79 trajectories) is significantly lower than QD-tagged GFP-GLT-1b-V5 (GLT-1b, 53 trajectories) at synaptic sites (P = 0.035, Mann-Whitney test).

**Supplementary Figure 4: Glutamate increases extrasynaptic GLT-1a surface diffusion in a transporter activity dependent manner**.

Hippocampal neuron-astrocytes mixed culture transfected with GFP-GLT-1a-V5 at DIV10 and imaged at DIV13. *(A)* Representative time lapse imaging illustrates GFP-GLT-1a-V5 in a region of an astrocyte (top panels) and overlaid by QD-tagged GFP-GLT-1a-V5 trajectories shown in orange (bottom panels). *(B)* Instantaneous diffusion coefficients, *(C)* Representative single trajectories, Scale bar, 0.5 μm, and MSDt plot of QD-tagged GFP-GLT-1a-V5. Control before treatment (black, median D= 0.071 μm^2^/s; n = 615 trajectories), after 2 min with 100 μM glutamate (red, median = 0.093 μm^2^/s; n = 557 trajectories), after drug washout (blue, median D= 0.072 μm^2^/s; n = 449 trajectories) and after 2 min of TFB-TBOA 10 μM+ glutamate 100 μM (green, median D= 0.070 μm^2^/s; n = 755 trajectories). Median D is significantly increased under glutamate (P = 0.002, Mann-Whitney test). Note MSDt exhibit linear profiles indicating free diffusion behaviour increasing with glutamate and decreasing with TFB-TBOA comparing to the control and after wash.

**Supplementary Figure 5: Glutamate increases extrasynaptic GLT-1b surface diffusion in a transporter activity dependent manner**.

Hippocampal neuron-astrocytes mixed culture transfected with GFP-GLT-1b-V5 at DIV10 and imaged at DIV13. *(A)* Representative time lapse imaging illustrates GFP-GLT-1b-V5 in a region of an astrocyte (top panels) and overlaid by QD-tagged GFP-GLT-1b-V5 trajectories shown in orange (bottom panels). *(B)* Instantaneous diffusion coefficients, *(C)* Representative single trajectories, Scale bar, 0.5 μm, and MSDt plot of QD-tagged GFP-GLT-1b-V5. Control before treatment (black, median = 0.074μm^2^/s; n = 783 trajectories), after 3min with 100 μM glutamate (red, median = 0.083μm^2^/s; n = 591 trajectories), after drug washout (blue, median = 0.070μm^2^/s; n = 494 trajectories) and after 3min of TFB-TBOA 10 μM+ glutamate 100 μM (gray, median = 0.063μm^2^/s; n = 571 trajectories). Median D is significant increased after 3min of 100 μM glutamate (P = 0.001, Mann-Whitney test) or significant decreased after 3min of TFB-TBOA 10 μM+ glutamate 100 μM (P=10.10^-6^, Mann-Whitney test) but is not signiﬁcantly different after drug wash (P > 0.05, Mann-Whitney test). Note MSDt exhibit linear profiles indicating free diffusion behaviour increasing with glutamate and decreasing with TFB-TBOA comparing to the control and after wash.

**Supplementary Figure 6: Neuronal activity bi-directionally regulates extrasynaptic GLT-1b surface diffusion in a transporter activity dependent manner**.

Hippocampal neuron-astrocytes mixed culture transfected with GFP-GLT-1b-V5 at DIV10 and imaged at DIV13. *(A and B)* Representative time lapse imaging illustrates GFP-GLT-1b-V5 in a region of an astrocyte (left panels) and overlaid by QD-tagged GFP-GLT-1b-V5 trajectories shown in orange (right panels). *(C, D)* Instantaneous diffusion coefficients of QD-tagged GLT-1b*,* *(C)* control untreated (black, median = 0.071 μm^2^/s; n = 613 trajectories), after 20min with 1mM 4-AP (red, median = 0.13 μm^2^/s; n = 1827 trajectories) and after 20min of 4-AP 1mM + TFB-TBOA 10 μM (blue, median = 0.070 μm^2^/s; n = 298 trajectories). Median D is significant increased after 20min with 1mM 4-AP (P = 4.5x10^-41^, Mann-Whitney test) but is not signiﬁcantly different after 20min of TFB-TBOA 10 μM+ 4-AP 1mM (P=0.5, Mann-Whitney test). *(D)* Control untreated (black, median = 0.071 μm^2^/s; n = 450 trajectories), after 20min with 1μM TTX (red, median = 0.053 μm^2^/s; n = 585 trajectories). Median D is signiﬁcantly different after 20min with 1μM TTX (P=9.6x10^-9^, Mann-Whitney test). *(E, F)* Mean squared displacement versus time, MSDt plot, of QD-tagged GLT-1b. Note MSDt exhibit linear profiles indicating free diffusion behaviour that increases with 4-AP *(E)* and decreases with TTX *(F)*.
